# Accuracy of plasma sTREM-1 for sepsis diagnosis in systemic inflammatory patients: a systematic review and meta-analysis

**DOI:** 10.1186/cc11884

**Published:** 2012-11-29

**Authors:** Youping Wu, Fei Wang, Xiaohua Fan, Rui Bao, Lulong Bo, Jinbao Li, Xiaoming Deng

**Affiliations:** 1 Department of Anesthesiology and Intensive Care, Changhai Hospital, Second Military Medical University, Shanghai, 200433, China

## Abstract

**Introduction:**

Early diagnosis of sepsis is vital to the clinical course and outcome of septic patients. Recently, soluble triggering receptor expressed on myeloid cells-1 (sTREM-1) appears to be a potential marker of infection. The objective of this systematic review and meta-analysis was to evaluate the accuracy of plasma sTREM-1 for sepsis diagnosis in systemic inflammatory patients.

**Methods:**

A systematic literature search of PubMed, Embase and Cochrane Central Register of Controlled Trials was performed using specific search terms (up to 15 October 2012). Studies were included if they assessed the accuracy of plasma sTREM-1 for sepsis diagnosis in adult patients with systemic inflammatory response syndrome (SIRS) and provided sufficient information to construct a 2 X 2 contingency table.

**Results:**

Eleven studies with a total of 1,795 patients were included. The pooled sensitivity and specificity was 79% (95% confidence interval (CI), 65 to 89) and 80% (95% CI, 69 to 88), respectively. The positive likelihood ratio, negative likelihood ratio and diagnostic odds ratio were 4.0 (95% CI, 2.4 to 6.9), 0.26 (95% CI, 0.14 to 0.48), and 16 (95% CI, 5 to 46), respectively. The area under the curve of the summary receiver operator characteristic was 0.87 (95% CI, 0.84 to 0.89). Meta-regression analysis suggested that patient sample size and assay method were the main sources of heterogeneity. Publication bias was suggested by an asymmetrical funnel plot (*P *= 0.02).

**Conclusions:**

The present meta-analysis showed that plasma sTREM-1 had a moderate diagnostic performance in differentiating sepsis from SIRS. Accordingly, plasma sTREM-1 as a single marker was not sufficient for sepsis diagnosis in systemic inflammatory patients.

## Introduction

Sepsis is a life-threatening complication of infection and the most common cause of death in intensive care units (ICU) [1]. Delay in diagnosis and treatment often results in rapid progression to circulatory collapse, multiple organ failure and eventual death [2]. Therefore, accurate and timely diagnosis of sepsis will limit morbidity, reduce costs and improve patients' outcome [3-5].

Diagnosis of sepsis is based on systemic inflammatory response syndrome (SIRS) in the presence of a known infection. SIRS is very common in critically ill patients, being found in various conditions, including trauma, surgery and pancreatitis [6,7]. Microbiological culture as a gold standard is used to distinguish sepsis from non-infectious conditions. However, this method lacks sensitivity, and there is often a substantial time delay. Thus, there is an urgent need for a fast, simple and accurate method to enhance sepsis diagnosis.

The triggering receptor expressed on myeloid cells-1 (TREM-1) was a recently discovered member of the immunoglobulin superfamily, expression of which on phagocytes was up-regulated by exposure to bacteria and fungi [8]. TREM-1 mediated the acute inflammatory response to microbial products. A soluble form of TREM-1 (sTREM-1) is released from the activated phagocytes and can be found in body fluids, such as plasma [9], pleural fluid [10], bronchoalveolar lavage fluid [11], urine [12] and cerebrospinal fluid [13]. Thus, sTREM-1 may act as a potential biomarker of bacterial infection [14,15]. Recently, several studies have been performed to investigate the role of plasma sTREM-1 in differentiating sepsis from non-infectious SIRS in different settings [9,16-25]. Due to the limited patient sample size recruited in the individual studies, we aimed to conduct a systematic review and meta-analysis to assess the role of plasma sTREM-1 for sepsis diagnosis in adult patients with SIRS.

## Materials and methods

This systematic review and meta-analysis was performed according to the guidelines of Meta-analysis of Observational Studies in Epidemiology [26].

### Search strategy

PubMed, Embase and Cochrane Controlled Clinical Trials Register Database (up to 20 June 2012) were searched by using Exploded Medical Subject Headings and the appropriate corresponding keywords, ''triggering receptor expressed on myeloid cells-1'', ''soluble triggering receptor expressed on myeloid cells-1'', ''TREM-1'', ''sTREM-1''. We updated the literature search of the above electronic databases on 15 October 2012 to find as many eligible studies as possible. No language restriction was used. Further searches were performed by checking the reference lists from primary and review articles, and manually reviewing abstract booklets and conference proceedings. The authors were contacted for study details if needed.

### Eligibility criteria and study selection

Studies were included if they assessed the accuracy of plasma sTREM-1 for sepsis diagnosis in adult patients with SIRS and provided sufficient information to construct a 2 X 2 contingency table. Two reviewers independently judged study eligibility when screening the citations. Disagreements were resolved by consensus. Agreement regarding study inclusion was assessed using the Cohen Κ statistic [27].

### Data extraction

Two reviewers independently abstracted data in each study to obtain information on the year of publication, country of origin, clinical setting, sample size, patients' demographics, sTREM-1 test methods, diagnostic cut-off points, sensitivity, specificity and methodological quality. Each reviewer extracted the data to construct a 2 X 2 contingency table.

### Definitions

Sepsis was defined according to the criteria proposed by the American College of Chest Physicians/Society of Critical Care Medicine as the presence of an infection complicated by SIRS [28]. Patients included in the septic group had either microbiologically (culture-proven) or clinically diagnosed sepsis, whereas the other patients were included in the non-infectious SIRS group.

### Quality assessment

The methodological quality of each study was graded independently by two reviewers with the Quality Assessment of Diagnostic Accuracy Studies (QUADAS) tool, a validated tool for the quality assessment of diagnostic accuracy studies [29]. Furthermore, studies were grouped according to Sackett and Haynes' [30] classification of diagnostic studies. In this classification, phase 1 studies are those that compare the difference in test results between patients with the target disorder and healthy individuals. Phase 2 studies are those that examine how the index test discriminates between patients with and without the target disorder. Phase 3 studies are those that assess the test's real-life performance in patients suspected of having the disorder.

### Statistical analysis

The diagnostic meta-analysis was performed using a bivariate meta-analysis model [31] to calculate the pooled sensitivity, specificity, positive/negative likelihood ratios, and diagnostic odds ratio (DOR). The summary receiver operator characteristic (SROC) curve that plotted sensitivity versus specificity was constructed to plot the individual and summary points of sensitivity and specificity [32]. Furthermore, around the pooled estimate, we also plotted a 95% confidence region and a 95% prediction region to illustrate the precision with which the pooled value was estimated (confidence ellipse of a mean) and to show the amount of between study variation (prediction ellipse; the likely range of values for a new study). The presence of statistical between-study heterogeneity was assessed by the I^2 ^test [33]. Values of 25, 50 and 75% for the I^2 ^test were regarded as indicative of low, moderate and high statistical heterogeneity, respectively. Meta-regression analysis using a bivariate model was performed in order to find the effect of potentially confounding covariates. Each covariate had a fixed effect when added to the bivariate model and associated with logit(sensitivity) and/or logit(specificity) [34]. Publication bias through small study effects was assessed with a regression test on the diagnostic odds ratio [35,36]. A *P-*value <0.05 was considered as indicative of statistical significance. Stata intercooled version 10.1 (StataCorp, College Station, TX, USA) was used for all statistical analyses.

## Results

### Study characteristics

**Figure 1:**
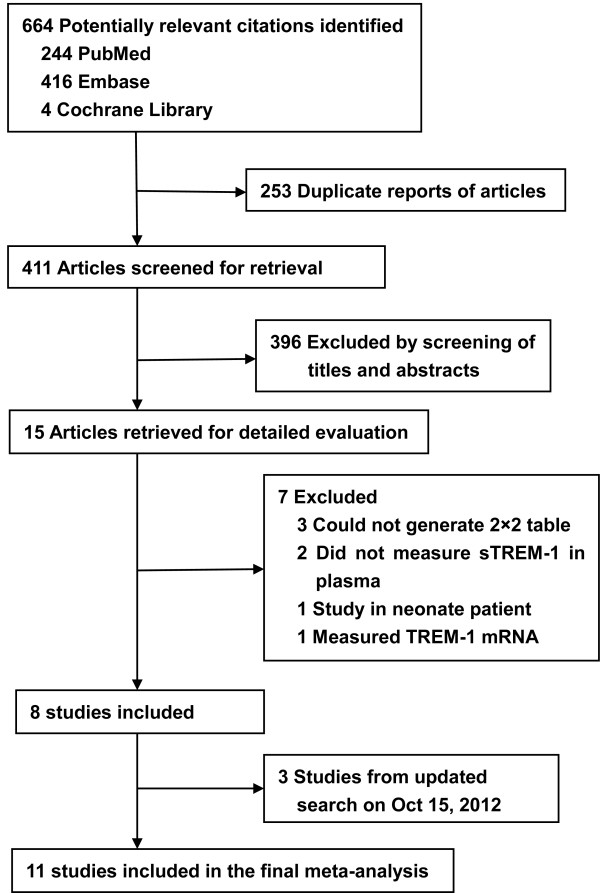
**Study identification, inclusion, and exclusion for meta-analysis**. Flow-chart of study selection.

The initial search yielded 664 citations, of which 15 publications dealing with sTREM-1 for sepsis diagnosis were considered as potentially suitable for inclusion. After full-text review, seven studies were excluded: three studies were excluded because the reviewers could not generate a 2 X 2 contingency table [37-39], one was excluded because it detected TREM-1 mRNA not sTREM-1 [40], two were excluded because it detected sTREM-1 in bronchoalveolar lavage fluid [41] or urine [12], and one was excluded because it targeted on neonates [42]. Three studies were included in the updated search (15 October 2012) [23-25]. Totally, 11 studies were included for the pooled analysis [9,16-25] (Figure 1). The Cohen Κ statistic for agreement on study inclusion was 0.92.

A total of 1,795 patients were included, comprising 933 patients from ICU in eight studies [9,18-21,23-25], and 862 patients from emergency departments in three studies [16,17,22]. Mean age varied from 31 to 69 years and the proportion of male ranged from 48% to 77%. The selected studies included a wide case mix, including trauma, medical and surgical illnesses. Among 1,795 patients included, SIRS criteria were fulfilled in 1,723 patients, including 1,076 septic patients and 647 non- infectious SIRS patients. Four studies recruited a group of well-matched (age and sex) patients without SIRS (10 patients in Soud *et al*.'s [16], 37 patients in Barati *et al*.'s [19], 10 patients in Giamarellos *et al*.'s [21], and 15 patients in Rivera *et al*.'s [20]) as control, and two studies [16,21] included the control patients as well as non- infectious SIRS patients in the non-septic group in a 2 X 2 contingency table when analyzing the diagnostic performance (that is, sensitivity and specificity) of sTREM-1 for sepsis. The prevalence of sepsis across studies ranged from 27% to 73%. To measure plasma sTREM-1 level, enzyme-linked immunosorbent assay (ELISA) was used in 10 studies, and Luminex multiplex assay in the 1 remaining study [22]. Among the included studies, the optimal cut-off point was retrospectively determined based on the receiver operating characteristic (ROC) curve and varied greatly, from 40 pg/ml to 755 pg/ml (ELISA method). Details of the included studies were shown in Table 1.

**Table 1:** Summary of included studies

Study year	Country	Setting	SIRS patients	Control patients	Mean age (year)	Assay method	Optimal timing	Cut-off (pg/ml)	Sensitivity/ specificity (%)	AUC	Sepsis prevalence (%)
**Group 1 studies**											
Giamarellos 2008 [21]	Greece	ICU	79 trauma patients with all criteria: (a) older than 18 years; (b) ISS greater than 25; (c) signs of SIRS	10 trauma patients with ISS greater than 25 but without SIRS	51.8 ± 20.6	ELISA (R&D Systems, Minneapolis, MN)	on admission	40	56.5/91.7	0.708	62
Soud 2011 [16]	Egypt	Surgical ED	80 trauma patients with SIRS	10 trauma patients with ISS greater than 25 but without SIRS	30.5	ELISA (R&D Systems, Minneapolis, MN)	Not reported	254	94.7/91.8	Not reported	27
**Group 2 studies**											
Barati 2010 [19]	Iran	medical and surgical ICUs	132 patients with SIRS	37 patients without SIRS	Not reported	ELISA (R&D Systems, Minneapolis, MN)	on admission	725	70/60	0.65	55
Gamez 2011 [17]	Colombia	ED	631 patients older than 18 years with any of the items: 1) suspected infection, 2) fever, 3) delirium, or 4) acute hypotension of unexplained origin within 24 hours of ED presentation	No	51 (36 to 68) ^†^	ELISA (R&D Systems, Minneapolis, MN)	within 24 hours after admission	135	60/59.2	0.614	66
Gibot 2004 [9]	France	medical ICU	76 patients with clinically suspected infection and SIRS	No	60 ± 15	ELISA (Dako, Glostrup, Denmark)	within 12 hours after admission	60	96/89	0.97	62
Gibot 2012 [25]	France	ICU	228 patients with clinically suspected infection	No	Not reported	ELISA (R&D Systems, Minneapolis, MN)	within 12 hours after admission	755	54.2/86.6	0.73	67
Kofoed 2007 [22]	Denmark	medical ED	151 patients with suspected community-acquired infections and SIRS	No	56 (20 to 94) ^†^	Luminex multiplex assay (Luminex Corp., Austin, TX)	on admission	3500	82/40	0.61	64
Latour 2010 [18]	Spain	two general ICU	114 patients older than 18 years with SIRS	No	Not reported	ELISA (R&D Systems, Minneapolis, MN)	within 24 hours after admission	463.2	49/79	0.62	63
Li 2012 [24]	China	surgical ICU	52 patients with clinically suspected infection and SIRS	No	55.7	ELISA (R&D Systems, Minneapolis, MN)	within 12 hours after admission	73.57	79/79	0.820	73
Rivera 2009 [20]	USA	surgical ICU	108 patients with clinically suspected infection and SIRS	15 trauma patients with ISS greater than 25 but without SIRS	35	ELISA (R&D Systems, Minneapolis, MN)	within 12-36 hours after admission	230	98/91	0.97	60
Su 2012 [23]	China	respiratory, surgical and emergency ICUs	144 older than 18 years with new fever and SIRS	No	54.5	ELISA (R&D Systems, Minneapolis, MN)	within 24 hours after admission	108.9	83/81	0.868	58

Group 1 and group 2 studies represented phase 2 and phase 3 studies according to Sackett and Haynes' classification for diagnostic studies, respectively. AUC, area under the receiver operating characteristic curve; ED, emergency department; ELISA, enzyme-linked immunosorbent assay; ICU, intensive care unit; ISS, injury severity score; SIRS, systemic inflammatory response syndrome. ^† ^Median (25th to 75th percentiles).

### Quality assessment

**Figure 2:**
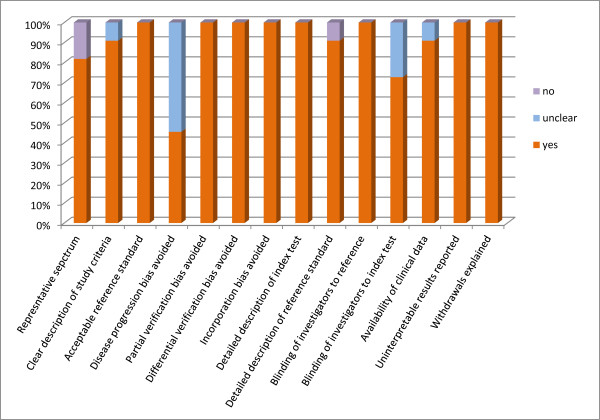
**Proportion of Quality Assessment of Diagnostic Accuracy Studies (QUADAS) tool criteria fulfilled for included studies**. Proportion of all 14 Quality Assessment of Diagnostic Accuracy Studies (QUADAS) tool criteria that were fulfilled for eleven studies included in the meta-analysis.

Studies were grouped according to Sackett and Haynes' classification for diagnostic studies: two were phase 2 studies (group 1) [16,21] and nine phase 3 studies (group 2) [9,17-20,22-25]. All the included studies fulfilled the requirements of acceptable reference standard, partial verification bias avoided, differential verification bias avoided, incorporation bias avoided, detailed description of index test, blinding of investigators to reference, uninterpretable results reported and withdrawals explained. Nine studies [9,17-20,22-25] recruited a representative spectrum of patients and 10 studies [9,16-24] clearly described the inclusion criteria. Disease progression bias was avoided in five studies [9,17,19,24,25]. Eight studies [9,17-20,22,24,25] fulfilled the requirement of blinding of investigators to index test. All except one [19] described in detail the reference standard and clinical data. The results of the methodological assessment for the included studies were summarized in Figure 2.

### Quantitative data synthesis

**Figure 3:**
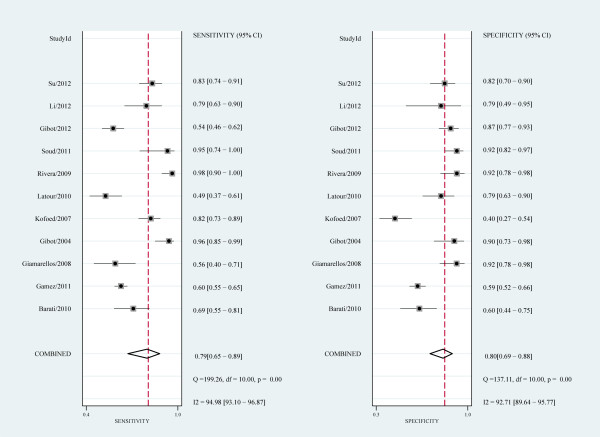
**Forrest plot of the sensitivity and specificity of sTREM-1 for the diagnosis of sepsis**. Forrest plot of the sensitivity and specificity of each individual study, pooled sensitivity and specificity, and I^2 ^statistic for heterogeneity.

**Figure 4:**
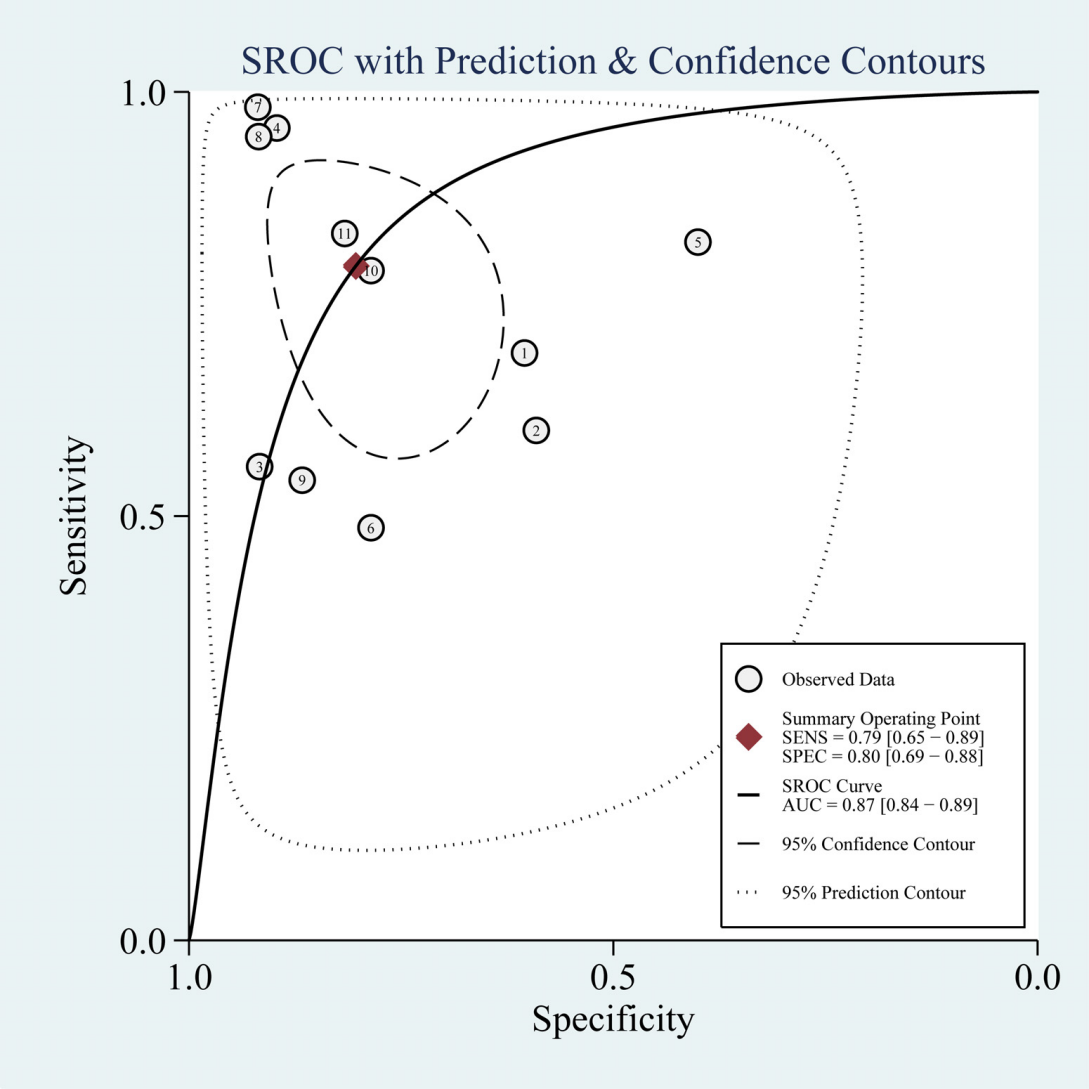
**Summary receiver operating characteristic graph of included studies**. Summary receiver operating characteristic graph with 95% confidence region and 95% prediction region for sTREM-1.

**Figure 5:**
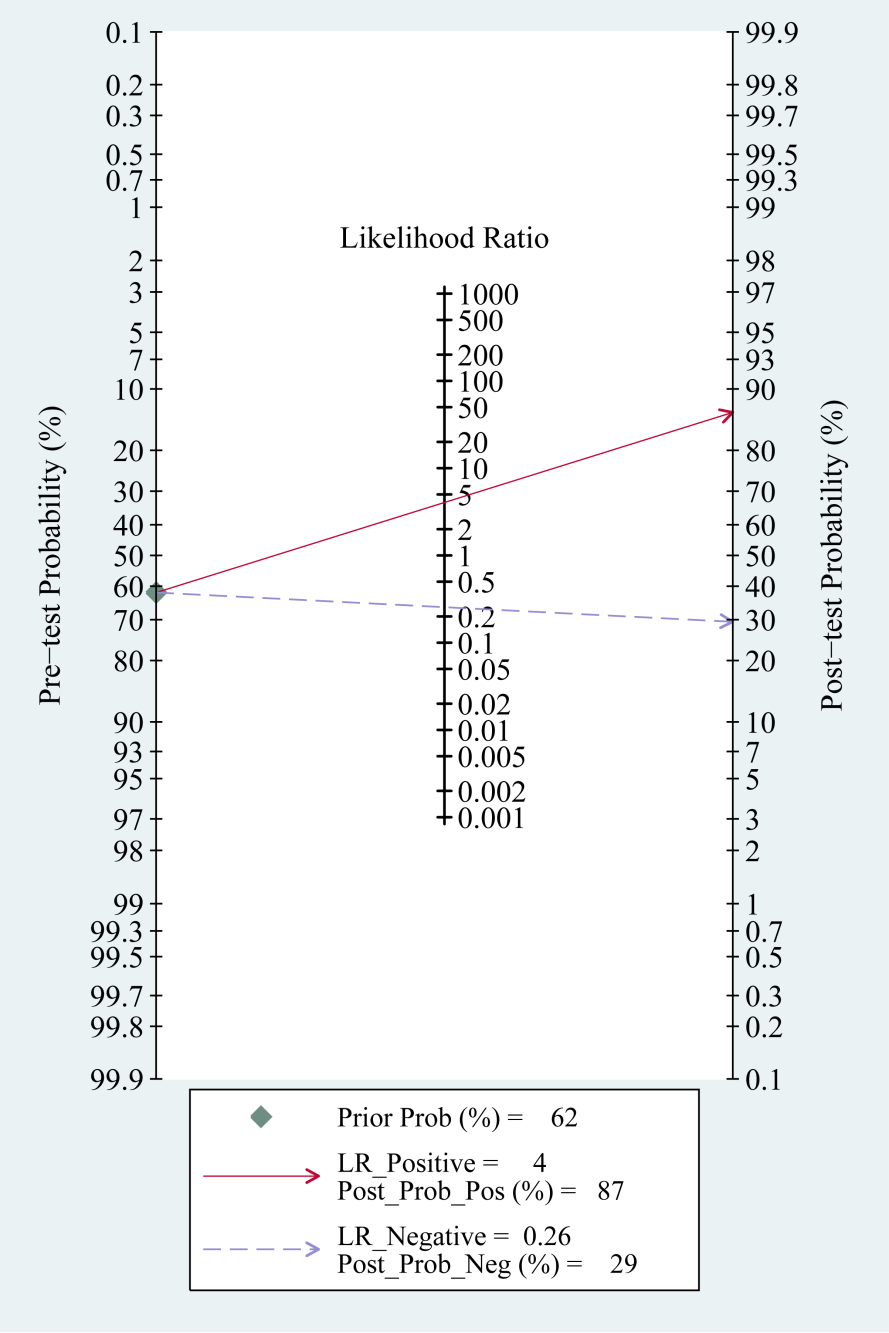
**Fagan's nomogram for calculation of post-test probabilities**. Fagan's nomogram for sTREM-1 illustrating post-test probability with a fixed pre-test probability of 62% for sepsis.

The pooled sensitivity and specificity of all studies combined was 79% (95% confidence interval (CI), 65 to 89) and 80% (95% CI, 69 to 88), respectively (Figure 3). The pooled positive likelihood ratio (PLR) was 4.0 (95% CI, 2.4 to 6.9) and the pooled negative likelihood ratio (NLR) was 0.26 (95% CI, 0.14 to 0.48). The area under the ROC curve was 0.87 (95% CI, 0.84 to 0.89) and the DOR was 16 (95% CI, 5 to 46), indicating a moderate diagnostic accuracy (Figure 4). The post-test probabilities based on various pre-test probabilities were illustrated using a Fagan nomogram (Figure 5).

Among the included studies, eight were conducted in the ICU and three in the emergency department. The pooled sensitivity and specificity was 78% (95% CI, 64 to 93) and 84% (95% CI, 76 to 93) in ICU patients, and 83% (95% CI, 63 to 100) and 68% (95% CI, 47 to 88) in patients from the emergency department. Nine studies [9,17-20,22-25] classified as phase 3 studies examined the diagnostic performance of plasma sTREM-1 for sepsis among SIRS patients suspected of infection, and the pooled sensitivity and specificity was 79% (95% CI, 66 to 92) and 76% (95% CI, 66 to 87), respectively. Given that Gamez *et al*.'s study [17] accounted for 35% (631/1,795) of the total sample size, sensitivity analysis without this study was conducted and the pooled sensitivity and specificity of the remaining 10 studies was 81% (95% CI, 70 to 92) and 82% (95% CI, 73 to 91).

### Investigation of heterogeneity

The between study variability (that is, heterogeneity) beyond what could be expected by sampling error was high both for sensitivity (with an I^2 ^of 95.0%) and for specificity (with an I^2 ^of 92.7%). The bivariate model analysis revealed that the heterogeneity was only partly (15%) explained by the threshold effect where variations in sensitivity and specificity were related to differences in the cut-off points of sTREM-1 in the included studies.

**Figure 6:**
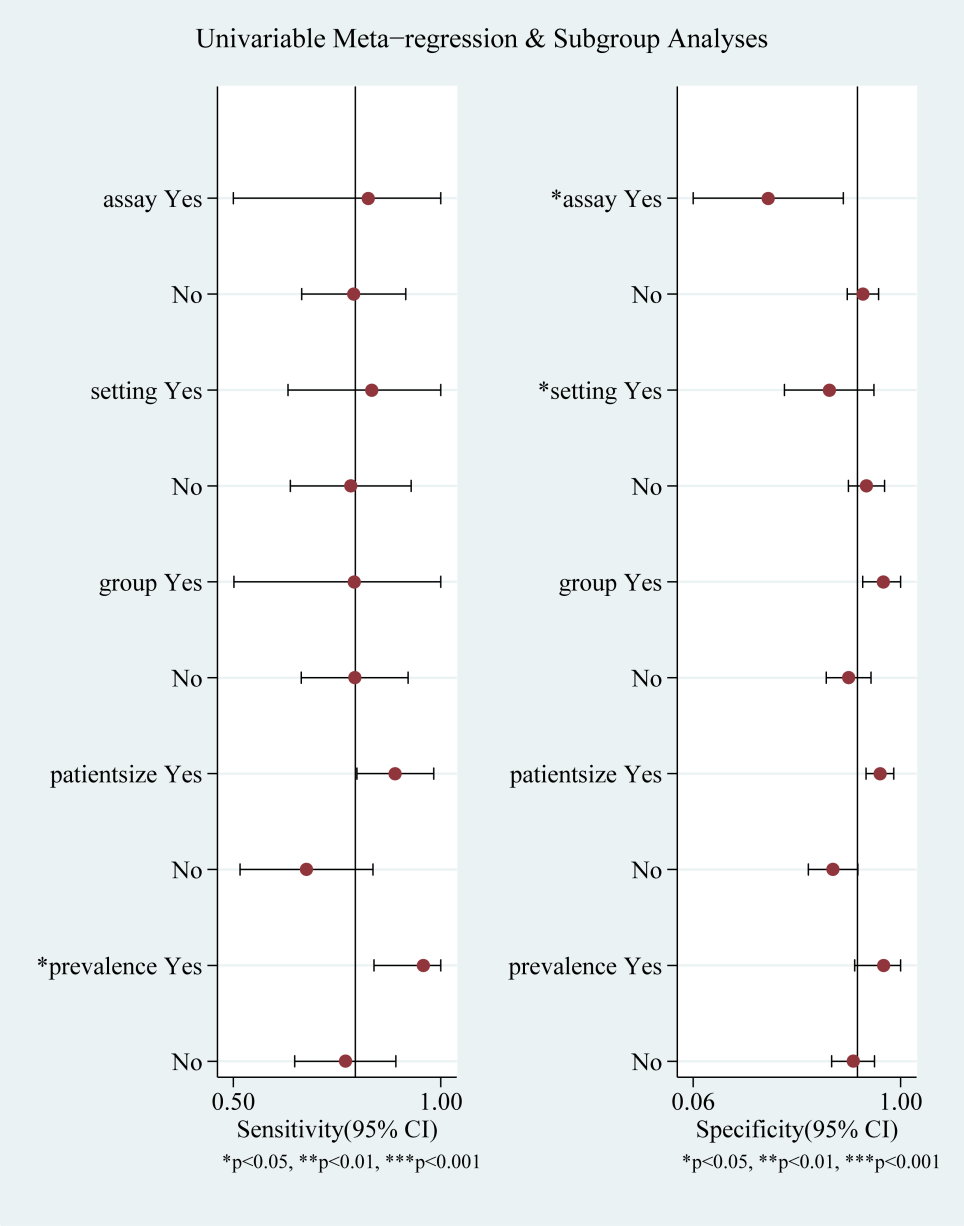
**Univariable meta-regression and subgroup analysis**.

The source of heterogeneity was explored by univariate meta-regression analysis. Assay method, clinical setting, quality assessment, patient sample size and sepsis prevalence were used as covariates. By making each covariate associate with logit(sensitivity) or logit(specificity), meta-regression analysis showed that sepsis prevalence significantly accounted for the heterogeneity for sensitivity, and assay method and clinical setting significantly accounted for the heterogeneity for specificity (Figure 6). When exploring the source of heterogeneity jointly by making each covariate associate with logit(sensitivity) and logit(specificity), meta-regression analysis showed that patient sample size and assay method were the main sources of heterogeneity. Subgroup analysis by patient sample size showed that in the five studies [9,16,20,21,24] with small sample size, the pooled sensitivity and specificity was 89% (95% CI, 80 to 98) and 91% (95% CI, 84 to 97) respectively, and in the other six studies [17-19,22,23,25] with large sample size, the pooled sensitivity and specificity was 68% (95% CI, 52 to 84) and 69% (95% CI, 58 to 81), respectively. Subgroup analysis by sTREM-1 assay method showed that Kofoed *et al*.'s study [22] using the Luminex multiplex assay had 83% sensitivity (95% CI, 50 to 100) and 40% specificity (95% CI, 6 to 74) and the other 10 studies using the ELISA had 79% sensitivity (95% CI, 67 to 92) and 83% pooled specificity (95% CI, 76 to 90).

### Evaluation of publication bias

**Figure 7:**
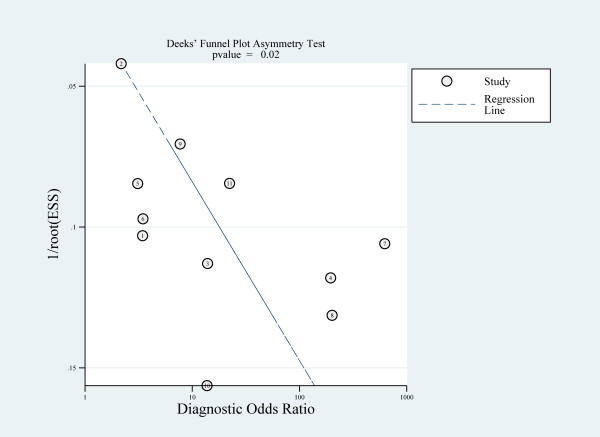
**Deeks' funnel plot asymmetry test for publication bias**.

Deeks' funnel plot asymmetry test suggested potential publication bias (*P *= 0.02) (Figure 7).

## Discussion

Early identification of infection is of vital importance to the clinical course and outcome of septic patients. An ideal marker of sepsis should be present early in the course of the disease, measurable rapidly and easily, of prognostic significance, sensitive enough to detect infection in patients with minimal host response, and specific enough to discriminate infection from other non-infectious SIRS [43].

Biological markers such as procalcitonin and CRP have been used in the diagnosis of bacterial infections [44,45]. However, since they are "indirect" markers of infection, their sensitivity and specificity are not 100% and vary in different patient groups and indications. TREM-1 was a recently identified molecule involved in inflammatory response. Human tissues infected with bacteria were infiltrated with neutrophils and monocytes that expressed high levels of TREM-1 [8,46]. TREM-1 amplifies infection-induced inflammatory response signals primarily through the mediation of adapter protein DAP12 on the cell surface [47]. sTREM-1, as the soluble form of TREM-1, released by activated phagocytes, may be a more "direct" marker of infection. In the present study, pooled analysis showed that plasma sTREM-1 seemed to have a moderate (0.7 ≤AUC <0.9) diagnostic accuracy for sepsis since the area under the SROC curve was 0.87. The pooled sensitivity of plasma sTREM-1 for the diagnosis of sepsis was 79% and the specificity was 80%. With a hypothetical pretest probability of 62% and a PLR of 4.0, detecting plasma sTREM-1 for sepsis diagnosis would raise the post-test probability to 87%. With a NLR of 0.26, detecting plasma sTREM-1 reduced the post-test probability to 30% (Figure 5), showing that application of plasma sTREM-1 test to sepsis diagnosis had a moderate value. Recently, several studies reported that plasma sTREM-1 level could be elevated in non-infectious disease, such as acute pancreatitis, and non-infectious inflammation following traumatic lung contusion [48-50]. We inferred that in the non-infectious SIRS patients, sTREM-1 level was elevated, which might partly account for its moderate but not high accuracy in distinguishing septic patients from non-infectious SIRS patients. To date, none of the proposed biomarkers as a single test had sufficient (more than 90%) sensitivity and specificity to discriminate sepsis from SIRS in critically ill adult patients. A combination of several markers appears to be a useful approach to improving accuracy in diagnosing sepsis, which was proved by a recent paper from Gibot *et al. *[25]. In their study, although sTREM-1, PCT and polymorphonuclear CD64 index were all found to be independent predictors of sepsis, a combination of them was shown to have a far better diagnostic performance for sepsis with the area under the ROC curve to be 97% (95% CI, 95 to 99).

As a single indicator of diagnostic test performance, DOR is independent of disease prevalence. The DOR of the included studies ranged from 2.17 to 623.33, and the pooled DOR was 16. The disparity noticed in the included studies may result from several reasons. First, patient sample size varied in the included studies. It was suggested that small studies tend to overestimate the effect size [51] and studies based on small sample sizes may have allowed for a type II error. In the present study, meta-regression analysis showed that patient sample size significantly accounted for the heterogeneity. The pooled DOR of the five studies [9,16,20,21,24] with small sample size was 90 (95% CI, 20 to 399), higher than that of the other six studies [17-19,22,23,25] with large sample size (pooled DOR 5, 95% CI, 3 to 9). Therefore, caution should be taken when interpreting the results. Another possible reason was different disease spectrum included. The pooled DOR of the three studies [16,20,21] which enrolled trauma or injured patients was 104 (95% CI, 9 to 1,206), higher than that of the remaining studies which enrolled other kinds of patient spectrum (pooled DOR 8, 95% CI, 3 to 19).

Two prior meta-analyses concluded that sTREM-1 represented a useful biological marker of bacterial infection [14] or bacterial pleural effusions [15]. The meta-analysis by Jing *et al. *[14] included studies across a wide range of disease spectrum, and assessed sTREM-1 level from different sample origins, including non-directed bronchial lavage fluids, pleural fluid, plasma, bronchoalveolar lavage fluid, cerebrospinal fluid and urine. The other meta-analysis by Summah *et al. *[15] assessed the diagnostic accuracy of sTREM-1 in the pleural fluid for bacterial pleural effusions. Being different from them, the present meta-analysis assessed the diagnostic accuracy of sTREM-1 for sepsis and focused only on the plasma level of sTREM-1. Although detecting the samples from suspected sites of infection might result in higher diagnostic accuracy than detecting the plasma sample, it is faster, easier and simpler to collect plasma samples.

The present meta-analysis had several limitations. First, although extensive literature search was conducted, the number of included studies was small. Second, in the patients who were clinically diagnosed as having sepsis without microbiological evidence, some degree of misclassification bias may have existed. Third, several studies reported sTREM-1 could reflect the severity of sepsis and predict prognosis, we didn't address this issue. Finally, we could not determine the ideal cut-off point for plasma sTREM-1 test because we did not have the raw data to map out the ROC curve. To determine whether there is a single threshold or a few important thresholds, further studies with a larger number of patients are needed.

## Conclusions

In summary, plasma sTREM-1 had a moderate diagnostic performance in differentiating sepsis from SIRS in adult patients. Accordingly, plasma sTREM-1 as a single marker was not sufficient for sepsis diagnosis in systemic inflammatory patients.

## Key messages

• The present study showed that the pooled sensitivity and specificity of plasma sTREM-1 for sepsis diagnosis was 79% and 80%, respectively. The area under the SROC curve was 0.87.

• Plasma sTREM-1 had a moderate diagnostic performance in differentiating sepsis from SIRS in adult patients. Accordingly, as a single marker it was not sufficient for sepsis diagnosis in systemic inflammatory patients.

## Abbreviations

AUC: area under the receiver operating characteristic curve; CI: confidence interval; DOR: diagnostic odds ratio; ELISA: enzyme-linked immunosorbent assay; ICU: intensive care unit; NLR: negative likelihood ratio; PLR: positive likelihood ratio; QUADAS: Quality Assessment of Diagnostic Accuracy Studies; ROC: receiver operating characteristic; SIRS: systemic inflammatory response syndrome; SROC: summary receiver operator characteristic; sTREM-1: soluble triggering receptor expressed on myeloid cells-1; TREM-1: triggering receptor expressed on myeloid cells-1.

## Competing interests

The authors declared that they have no competing interests.

## Authors' contributions

YW, FW, XF, JL and XD were responsible for study concept and design. YW, FW and XF acquired the data. RB and LB analyzed and interpreted the data. YW, FW and XF drafted the manuscript. JL and XD critically revised the manuscript for important intellectual content. YW and FW performed statistical analysis. RB and LB were responsible for administrative, technical and material support. JL and XD supervised the study. All authors have read and approved the manuscript for publication.
